# On a Key-Based Secured Audio Data-Hiding Scheme Robust to Volumetric Attack with Entropy-Based Embedding

**DOI:** 10.3390/e21100996

**Published:** 2019-10-12

**Authors:** Jose Juan Garcia-Hernandez

**Affiliations:** Cinvestav Unidad Tamaulipas, Km. 5.5 Carr. a Soto la Marina, 87130 Cd Victoria, Tamps., Mexico; jjuan@tamps.cinvestav.mx; Tel.: +52-834-1070251

**Keywords:** audio data-hiding, entropy, integer DCT, key-secured

## Abstract

In the data-hiding field, it is mandatory that proposed schemes are key-secured as required by the Kerckhoff’s principle. Moreover, perceptual transparency must be guaranteed. On the other hand, volumetric attack is of special interest in audio data-hiding systems. This study proposes a data-hiding scheme for audio signals, which is both key-based secured and highly perceptually transparent and, thus, robust to the volumetric attack. A modification to a state-of-the-art data-hiding algorithm is proposed to achieve key-based security. Embedding is carried out in the integer discrete cosine transform (DCT) domain; selected samples for embedding are determined by the entropy of the Integer DCT coefficients. Of the two key-based improvements proposed, the multiplicative strategy gives better results, guaranteeing the worst bit error rate when an incorrect key is used. Additionally, the perceptual transparency of the proposed scheme is higher, compared to the state-of-the-art schemes using similar embedding strategies.

## 1. Introduction

The rapid growth of big data facilities and communications services has democratized multimedia contents access. However, the ease of access has also caused problems, such as the illegal copying and distribution of copyrighted works. Digital watermarking has been used as a tool for copyright protection, data authentication, copy protection, traitor tracing, broadcast monitoring, and so on. Digital watermarking consists of embedding some information, known as a watermark, in an imperceptible and secure fashion in the original media, to show ownership, authenticate the multimedia, establish a secret communications channel, and so on. Watermark embedding is mainly carried out in two domains: the spatial/time domain (for images and video and audio, respectively) [1,2,3,4] or the frequency domain [5,6,7]. In audio watermarking, one of the most simple but effective attacks is the gain attack, which consists of varying the original volume uniformly to confuse the watermark detection/extraction algorithm. Spread spectrum (SS) watermarking [7,8] has shown robustness to volume attacks; however, this robustness is paid for by low embedding capacity and auditive transparency. On the other hand, quantization-based watermarking algorithms [9,10] have high embedding capacities and low distortion, thus they fail under volume attack.

An gain-invariant high-rate watermarking algorithm, known as Rational Dither Modulation (RDM), was proposed in [11]. RDM has been utilized for audio watermarking with good results [12]. Although RDM is one of the most relevant gain-invariant algorithms, the high peak-to-average power ratio of RDM is its main drawback [13]. Some studies have proposed improvements to RDM, which showed high-rate and gain-invariant properties [14,15,16]. Recently a gain-invariant watermarking algorithm was proposed in [13]; this algorithm showed high robustness against both uniform and variable gain attack. The algorithm in [13] was evaluated on digital images, showing robustness to gain attacks and typical digital signal processing operations, such as filtering, Gaussian noise addition, and lossy compression. However, despite its otherwise remarkable characteristics, this algorithm lacks key-based security. According to Kerckhoff’s principle, the security of a cryptosystem must be guaranteed only with the knowledge of the key, even if everything about the system is public. Although a watermarking system is not a cryptosystem in the whole sense, Kerckhoff’s principle is valid for the watermarking purposes.

In this paper, two strategies to achieve key-based security for the algorithm in [13] are proposed and evaluated. The resulting algorithm is used for audio watermarking in the integer Cosine Discrete Transform (intDCT) domain, using the entropy of intDCT coefficients as an embedding suitability indicator. The embedding is carried out in the high-entropy sub-bands in order to achieve good perceptual transparency. Entropy has been used as a selection criterion for data embeddings in an adaptive fashion [17,18,19,20,21], but mostly in the wavelet domain; to the best of our knowledge, this is the first time that the entropy of intDCT coefficients has been used for data embedding.

This paper is organized as follows. Section 2 introduces the related background. The security strategies for the algorithm in [13] are described in Section 3. Then, Section 4 introduces the proposed audio data-hiding algorithm. Experiments and results are presented in Section 5. Finally, Section 6 concludes the paper through a discussion.

## 2. Related Background

### 2.1. Entropy

Shannon and Wiener suggested a measure of uncertainty or entropy associated with the sample space of a complete finite scheme as follows,

(1) $$H\left(X\right)=-\sum_{i=1}^{n}p_{i}\log p_{i},$$

where $p_{i}$ is the probability of the occurrence of the event $E_{i}$, as described in Equations (2) and (3) [22].

(2) $$\left[E\right]=\left[E_{1},E_{2},\ldots ,E_{n}\right]$$

with $\cup_{k=1}^{u}E_{k}=U$, and

(3) $$\left[P\right]=\left[p_{1},p_{2},\ldots ,p_{n}\right]$$

with $\sum_{k=1}^{n}p_{k}=1$.

In this study, entropy is used as the selection criterion for data embedding.

### 2.2. Integer Discrete Cosine transform

The intDCT domain is used both for the embedding and extraction of the watermark. The forward DCT-IV transform of an *N*-point audio signal x[n] is given by Equation (4), and its inverse transform is given by Equation (6):

(4) $$\begin{array}{cc} X[m] & =C_{N}^{IV}\cdot x\left[n\right],\\ m=n & =0,1,\cdots ,N-1 \end{array}$$

where **X** represents the intDCT coefficients of **x** and $C_{N}^{IV}$ is the transform matrix, defined by

(5) $$C_{N}^{IV}=\sqrt{\frac{2}{N}}\left[\cos\left(\frac{\left(m+\frac{1}{2}\right)\left(n+\frac{1}{2}\right)\pi }{N}\right)\right],$$

where m=0,1,⋯,N−1 and n=0,1,⋯,N−1. As $C_{N}^{IV}$ is an orthogonal matrix, and the inverse intDCT transform is given by

(6) $$x\left[n\right]=C_{N}^{IV}\cdot X\left[m\right].$$

The intDCT is used in this study because the embedding and extraction algorithms require an integer representation of the frequency components of the signal to calculate the entropy. In this implementation, the fast intMDCT algorithm, proposed by the authors of [23], is used to calculate the intDCT, which is an approximation of the DCT-IV. The fast intMDCT algorithm divides the transform matrix into five submatrices; multiplication by each of these five submatrices is done through a lifting stage with a rounding operation. The intDCT coefficients are obtained through the five lifting stages.

### 2.3. Peak Signal-to-Noise Ratio

The peak signal-to-noise ratio (PSNR) measures the similarity of two signals—typically a reference signal and a processed version of it—which defines the relation between the maximum energy of a signal and the noise affecting it, expressing this ratio in decibels [24,25]. Given a 16-bit audio clip *f* and a copy of the processed audio *g*, both of size *N*, the PSNR between *f* and *g* is computed by

(7) $$\mathrm{PSNR}(f,g)=10\cdot \log_{10}\left(\frac{65,535^{2}}{MSE(f,g)}\right),$$

(8) $$MSE\left(f,g\right)=\frac{1}{N}\sum_{i=1}^{N}{\left(f_{i}-g_{i}\right)}^{2}.$$

As for the mean square error (MSE), the difference between the samples $f_{i}$ and $g_{i}$ is considered to be an error that generates signal quality loss. The lower the MSE, the higher the PSNR; therefore, the higher the PSNR (f, g) values, the higher the signal quality. For audio and image signals, a PSNR greater than 35 dB is typically considered of good quality.

## 3. Key-Based Security Strategies for the Gain-Invariant Algorithm by Zareian and Tohidypour

### 3.1. The Gain-Invariant Algorithm

In [13], a gain-invariant algorithm was proposed under the quantization index modulation (QIM) data-hiding paradigm. In this Section, this algorithm is detailed, as follows.

#### 3.1.1. Data Embedding

Let be $u=\left[x_{1},x_{2},\ldots ,x_{N}\right]$ be the *N*-sample host signal. Then, u is splitted into two sequences: x, containing even index samples, and y containing the odd index samples (i.e., $x_{i}=u_{2i}$ and $y_{i}=u_{2i-1}$ with $i=1,\ldots ,\frac{N}{2}$). The message, m∈{0,1}, is embedded in u using the $l_{p}$-norm of x and y, as follows.

First, $l_{x}$ and $l_{y}$ are calculated as

(9) $$l_{x}={\left(\frac{2}{N}\sum_{i=1}^{\frac{N}{2}}{\left|u_{2i}\right|}^{p}\right)}^{\frac{1}{p}}\;\;\mathrm{and}\;\;\;\;\;\;l_{y}={\left(\frac{2}{N}\sum_{i=1}^{\frac{N}{2}}{\left|u_{2i-1}\right|}^{p}\right)}^{\frac{1}{p}},$$

where $l_{x}$ and $l_{y}$ are the $l_{p}$-norms of x and y, respectively, and p≥1. The ratio $z=\frac{l_{x}}{l_{y}}$ is calculated and used as the host of a QIM embedding, as follows,

(10) $$z_{q}=Q_{m}\left(z\right)=\Delta round\left(\frac{z+m\Delta /2}{\Delta }\right)-m\frac{\Delta }{2},m\in \left\{0,1\right\},$$

where Δ is the quantization step of QIM.

Next, the subsequences x and y are updated:

(11) $$x_{i}^{\prime }=\sqrt{\frac{z_{q}}{z}}x_{i}\;\;,\;\;\;\;\;\;y_{i}^{\prime }=\sqrt{\frac{z_{q}}{z}}y_{i}.$$

Finally, the watermarked signal $u^{\prime }$ is obtained by repositioning $x^{\prime }$ and $y^{\prime }$ in the even and odd positions, respectively.

#### 3.1.2. Data Extraction

For extraction, the received signal $u^{\prime }$ is divided into two subsequences, in the same fashion as when embedding: $x^{\prime \prime }$ and $y^{\prime \prime }$ contain the even and odd samples positions, respectively. Then, the respective $l_{p}$-norms are calculated as

(12) $$l_{x}^{\prime \prime }={\left(\frac{2}{N}\sum_{i=1}^{\frac{N}{2}}{\left|u_{2i}^{\prime \prime }\right|}^{p}\right)}^{\frac{1}{p}}\;\;\mathrm{and}\;\;\;\;\;\;l_{y}^{\prime \prime }={\left(\frac{2}{N}\sum_{i=1}^{\frac{N}{2}}{\left|u_{2i-1}^{\prime \prime }\right|}^{p}\right)}^{\frac{1}{p}}.$$

Finally, under the QIM paradigm, the data is extracted as follows,

(13) $$\hat{m}=\arg\min_{m\in \{0,1\}}\left|z^{\prime \prime }-Q_{m}\left(z^{\prime \prime }\right)\right|,$$

where $z^{\prime \prime }=\frac{l_{x}^{\prime \prime }}{l_{y}^{\prime \prime }}$. The decoder only needs to know Δ for data extraction.

### 3.2. The Additive Strategy for Key-Based Security

The algorithm proposed in [13] lacks key-based security; although the Δ value could be considered a kind of secret key, it would be impractical, as an arbitrary Δ value could not be optimum according to the analysis and performance evaluation reported in Section 3 of [13].

In this paper, we propose two strategies to provide security based on an arbitrary key: The first is called the additive strategy and consists of adding a key-generated PN-sequence to the host signal prior to data embedding, similar to strategy reported in [26]; then, the PN-sequence of the watermarked signal is subtracted. The PN-sequence αn with n∈{−1,1}, where α is the distortion factor, which is generated using the same key as the PN generator seed.

For extraction, the same PN-sequence is generated using the secret key and added to the received watermarked signal; then, the extraction algorithm is applied. This additive strategy sets a symmetric-key scenario.

**Figure 1:**
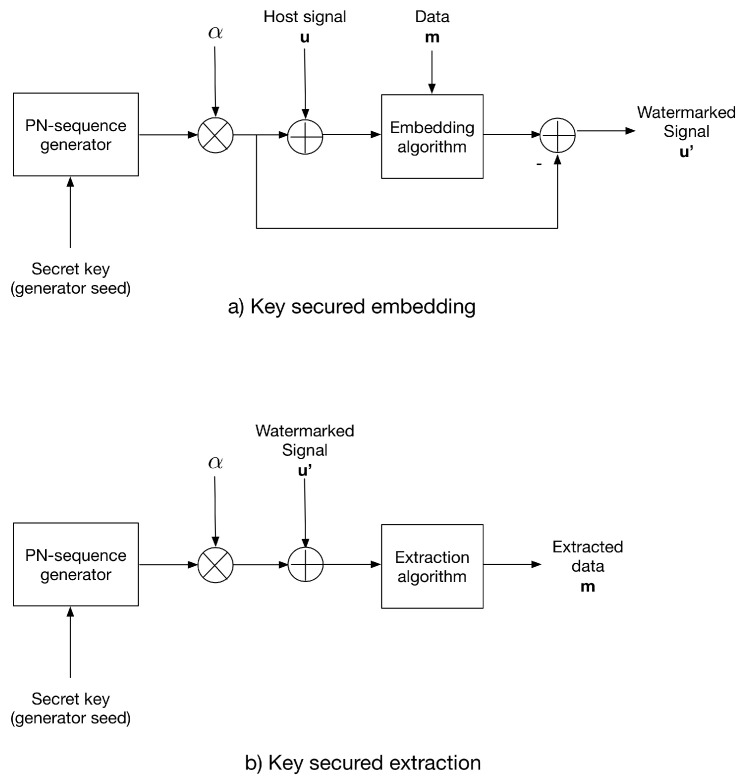
The additive strategy for (**a**) data embedding and (**b**) data extraction.

Figure 1 shows the additive strategy conception.

### 3.3. The Multiplicative Strategy for Key-Based Security

The multiplicative strategy modulates the host signal using a key-generated PN-sequence prior to the embedding process. Same to additive strategy, the PN-sequence αn with n∈{−1,1}, where α is the distortion factor, generated using the same key as the PN generator seed. After data embedding, the watermarked signal is divided by the PN-sequence.

For extraction, the same PN-sequence is generated using the secret key and used to modulate the received watermarked signal; then, the extraction algorithm is applied.

**Figure 2:**
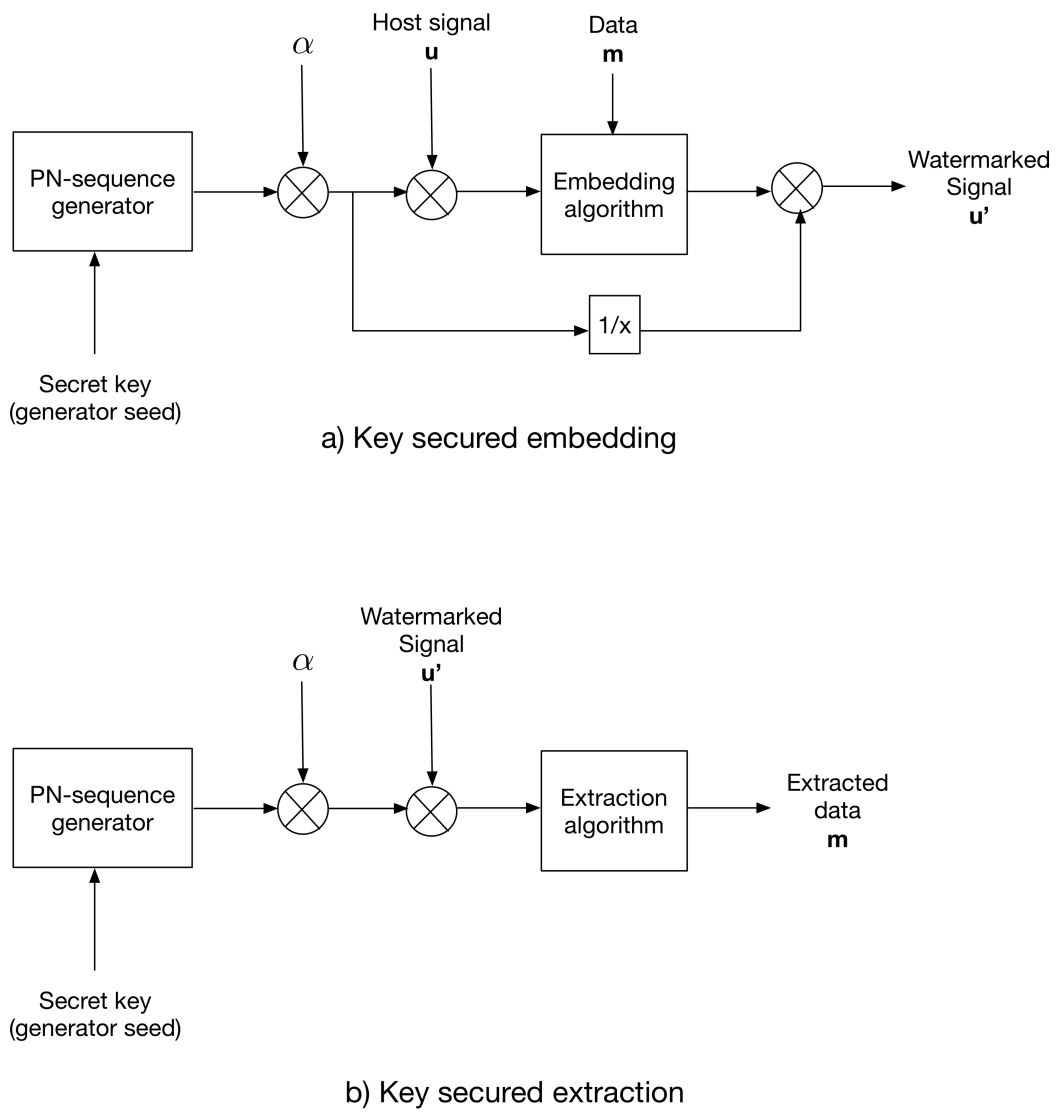
The multiplicative strategy for (**a**) data embedding and (**b**) data extraction.

Figure 2 shows the multiplicative strategy conception.

## 4. The Proposed Watermarking Scheme

This section introduces the proposed audio watermarking scheme, divided into the insertion process and the extraction process.

### 4.1. The Insertion Process

The data embedding is carried out as follows.

- The audio signal is segmented into *N*-sample blocks and intDCT transformed.
- Each transformed audio block is splitted into eight sub-bands. This number of sub-bands was experimentally determined, to achieve a trade-off between auditive transparency and payload, as each sub-band is a potential carrier of one bit.
- Transformed sub-bands are divided by $2^{8}$; the CD-quality audio dynamic range is $\left[2^{-15},2^{15}-1\right]$, which for a small set of samples, entropy computation will lead the same entropy value at any time, $\log\frac{N}{8}$, as the sub-band distributions will be uniform. This situation applies for both audio blocks and transformed blocks, as intDCT is a linear transform.
- For each sub-band in a transformed block, entropy is computed; then, the average entropy of all sub-bands is computed and set as the embedding threshold for that block in a similar manner as in [21].
- Data is embedded into sub-bands showing a higher entropy than the threshold; embedding is carried out using one of the strategies described in Section 3.
- Finally, the watermarked audio block is returned to the time domain by the inverse intDCT, and thus the watermarked audio is obtained.

**Figure 3:**
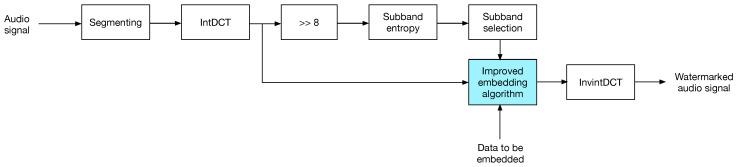
The watermark insertion process.

Figure 3 shows the insertion process.

### 4.2. The Extraction Process

For data extraction, most of the steps described for embedding in Section 4.1 are followed:

- Audio signal is segmented in *N*-samples blocks and intDCT transformed.
- Each transformed block is splitted into eight sub-bands.
- Transformed sub-bands are divided by $2^{8}$.
- For each sub-band in a transformed block, entropy is computed; then, the average entropy of all sub-bands is computed.
- Data is extracted from sub-bands showing a higher entropy than threshold using the corresponding strategy as described in Section 3.

**Figure 4:**
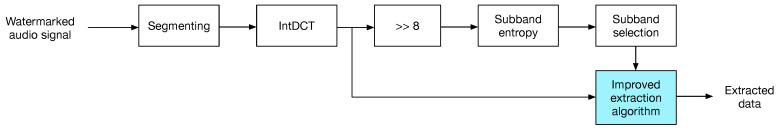
The watermark extraction process.

Figure 4 shows the insertion process.

## 5. Experiments and Results

This section describes the experimental setup and results in this study. For all of the experiments, the audio block length *N* was set to 4096; Δ and *p* were set to the optimal values found in [21] (0.087 and 2.1, respectively).

### 5.1. Audio Dataset and Computing Platform

The audio dataset consisted of 982 CD-quality audio clips; each clip had a sampling rate of 44,100 samples per second, had one channel, and was 5 s long. All of clips were musical, from different music styles ranging from classical to big band and including Latin pop and Caribbean rhythms; no music style classification was explicit (The dataset is fully available in [27]). All experiments were carried out using the Matlab R2014b software (Mathworks, Natick, MA, USA) running on a workstation with an Intel Xeon processor at 3.2 GHz and 32 GB of RAM.

### 5.2. Auditive Transparency

The proposed data-hiding scheme was evaluated in terms of PSNR and an audio transparency metric known as the Objective Difference Grade (ODG) [28]. ODG has values in [0,−4], with an ODG value between 0 and −1 indicating a very good perceptual transparency [28]. Note that the free basic implementation of the PEAQ algorithm [29] was used to obtain the ODG results; therefore, they were not exactly in the range [0,−4]. In this section, the distortion results of data-hiding are given for both unsecured and secured data-hiding. In addition, concerning the distortion (PSNR and ODG) results, a statistical significance analysis was conducted by means of the Kruskal–Wallis test (significance level = 0.05), to evaluate whether the medians of the compared results were different [30]. Additionally, correction for multiple testing on the basis of the same data was made using the Bonferroni correction [31].

#### 5.2.1. Unsecured Data-Hiding

**Figure 5:**
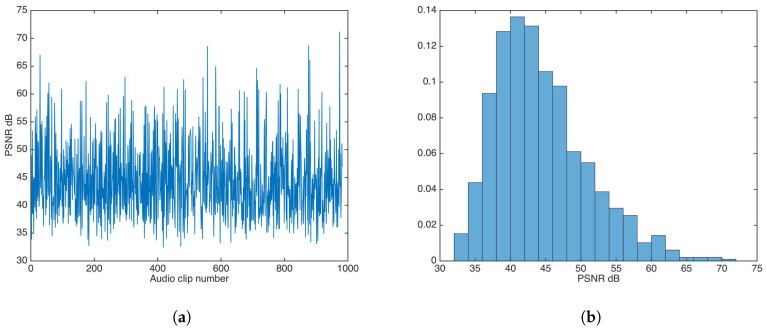
Peak signal-to-noise ratio (PSNR) results for watermarking without key-based security. (**a**) PSNR results. (**b**) PSNR distribution.

The whole audio dataset [27] was fed to the watermark insertion process without any key-based security strategy. Figure 5 shows the PSNR results and PSNR distribution in this experiment.

The PSNR mean and median were calculated to be 44.37 and 43.29, respectively; both values are very good for practical applications.

**Figure 6:**
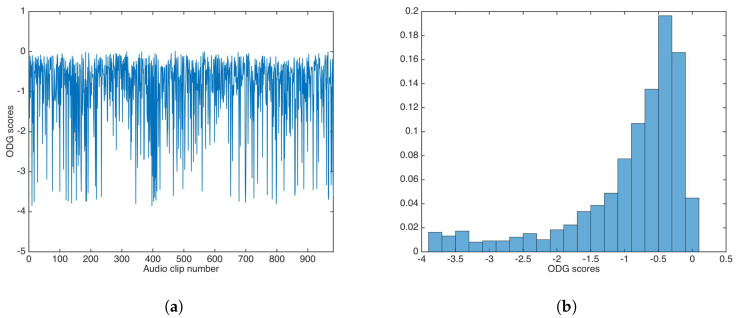
Objective Difference Grade (ODG) results for watermarking without key-based security. (**a**) ODG results. (**b**) ODG distribution.

ODG evaluation was carried out in similar way for the audio dataset. Figure 6 shows the ODG results and the ODG distribution in this experiment.

As with PSNR, ODG mean and median were calculated as −0.9362 and −0.6318, respectively; again, both values are very good for practical applications.

#### 5.2.2. Key-Based Additive Strategy

**Figure 7:**
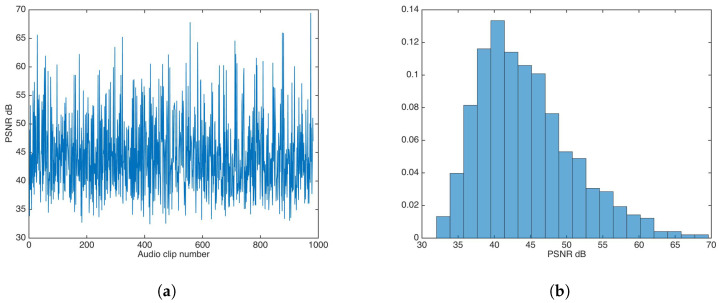
PSNR results for watermarking using the additive strategy. (**a**) PSNR results. (**b**) PSNR distribution.

The additive strategy was evaluated for α values $\in [\frac{1}{16}\ldots 1]$, in steps of $\frac{1}{16}$ in descending fashion, until the highest α value was achieved which approximated the distortion results for the unsecured data-hiding version. The found α value was $\frac{1}{8}$; Figure 7 shows the PSNR results and the PSNR distribution for that α value.

The PSNR mean and median were calculated as 44.36 and 43.30, respectively.

**Figure 8:**
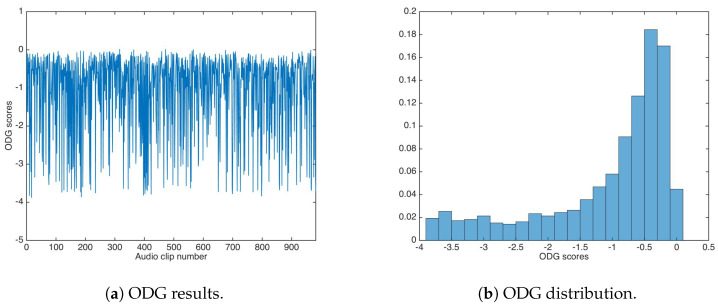
Objective Difference Grade (ODG) results for watermarking using the additive strategy.

The ODG evaluation achieved similar values as the unsecured data-hiding version; Figure 8 shows the ODG results and the ODG distribution. The ODG mean and median were calculated as −1.0638 and −0.6409 respectively.

On the other hand, statistical tests shown no statistical difference, in terms of PNSR and ODG, for the additive strategy.

#### 5.2.3. Key-Based Multiplicative Strategy

**Figure 9:**
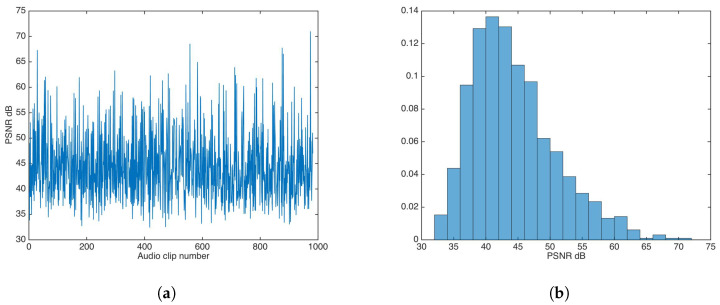
PSNR results for watermarking using the multiplicative strategy. (**a**) PSNR results. (**b**) PSNR distribution.

In the same fashion as the additive strategy, the multiplicative strategy was evaluated for α values $\in [\frac{1}{4}\ldots 2]$, in steps of $\frac{1}{4}$ in descending fashion, until the highest α value was achieved that approximated the distortion results for the unsecured data-hiding version. The found α value was 1; Figure 9 shows the PSNR results and the PSNR distribution for that α value.

The PSNR mean and median were calculated as 44.34 and 43.29, respectively.

**Figure 10:**
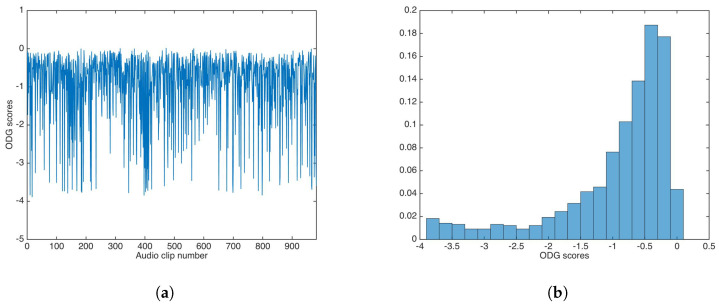
ODG results for watermarking using the multiplicative strategy. (**a**) ODG results. (**b**) ODG distribution.

The ODG evaluation achieved similar values as the unsecured data-hiding version; Figure 10 shows the ODG results and the ODG distribution. The ODG mean and median were calculated as −0.9387 and −0.6222, respectively.

For the multiplicative strategy, the statistical tests showed no statistical differences in terms of PNSR and ODG. Similar to the unsecured data-hiding version, the multiplicative PSNR and ODG results are very good for practical applications.

### 5.3. Key-Based Security

In this section, the effectiveness of both additive and multiplicative strategies is evaluated in terms of Bit Error Rate (BER). It is well-known that the most effective security mechanism will produce BER values equal to 0.5 when an incorrect key is used. According to Information Theory, no practical Error Correcting Codes exist for BER = 0.5, as Repetition Codes become effective for that BER for infinite codeword lengths. Therefore, a key-based security strategy producing BER = 0.5 is mandatory.

#### 5.3.1. Key-Based Additive Strategy

Using the additive strategy, the whole audio set was watermarked utilizing a key, $k_{1}$. Then, data was extracted utilizing a key $k_{2}$ with $k_{1}\neq k_{2}$. As detailed above, the α value was set to $\frac{1}{8}$.

**Figure 11:**
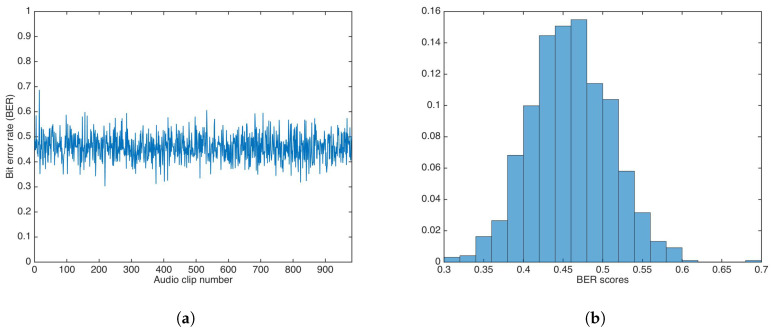
Bit Error Rate (BER) results for watermarking using the additive strategy and incorrect key. (**a**) BER results. (**b**) BER distribution.

Figure 11 shows the BER results and the BER distribution for the additive strategy.

The BER mean, median, and variance were calculated as 0.4591, 0.4582, and 0.0025, respectively. Both the mean and median are not equal to 0.5.

#### 5.3.2. Key-Based Multiplicative Strategy

Using the multiplicative strategy, the whole audio set was watermarked utilizing a key $k_{1}$. Then, data was extracted utilizing a key, $k_{2}$, with $k_{1}\neq k_{2}$. The α value was set to 1.

**Figure 12:**
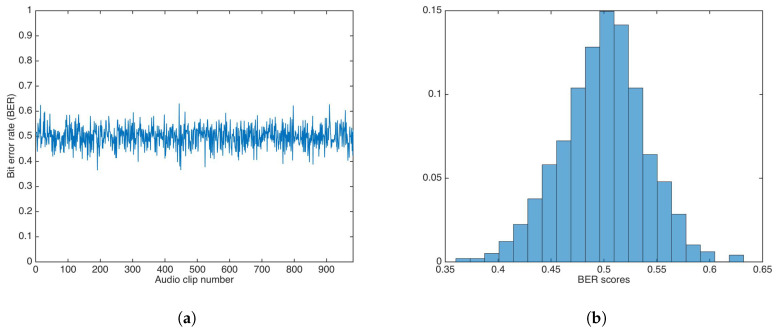
BER results for watermarking using the multiplicative strategy and incorrect key. (**a**) BER results. (**b**) BER distribution.

Figure 12 shows the BER results and the BER distribution for the multiplicative strategy.

The BER mean, median, and variance were calculated as 0.4993, 0.5000, and 0.0016, respectively. Both mean and median were equal (or very close) to 0.5.

A Wilcoxon rank-sum test was carried out to determine if both methods showed the same behavior. The test rejection of the null hypothesis of equal median at the default 5% significance level (p=5.9499e−75) indicated that the multiplicative strategy was better than additive strategy, as the median and mean in the multiplicative strategy were closer to equal to 0.5, the ideal BER value for an incorrect key.

### 5.4. Statistical Transparency

**Figure 13:**
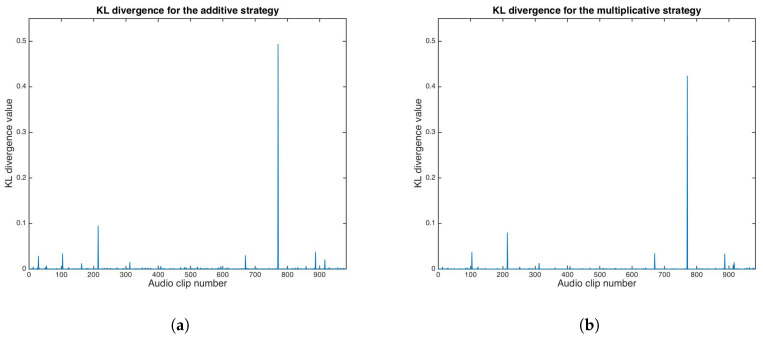
Kullback–Leibler divergence results. (**a**) Additive strategy. (**b**) Multiplicative strategy.

In this section, the statistical transparency of the proposed system is evaluated in terms of Kullback–Leibler (KL) divergence. KL divergence between probability distributions $P_{Q_{0}}$ and $P_{Q_{1}}$ is defined as follows,

(14) $$D(P_{Q_{0}}\left|\right|P_{Q_{1}})=\sum_{q\in Q}P_{Q_{0}}\left(q\right)\log\frac{P_{Q_{0}}\left(q\right)}{P_{Q_{1}}\left(q\right)}$$

with $0\log\frac{0}{0}=0$ and $p\log\frac{p}{0}=\infty$ if p>0. According to the authors of [32], a steganographic system is called ϵ−*secure* if

(15) $$D(P_{C}||P_{S})\leq \epsilon$$

where $P_{C}$ is the distribution of *C*, $P_{S}$ is the distribution of *S*, and ϵ is an arbitrary low number. If ϵ=0, the steganographic system is a “perfectly secure” case. However, very low ϵ values are enough for steganographic systems for real-world signals. Figure 13 shows KL divergence results for both the additive and multiplicative strategies.

KL divergence median values for the additive and multiplicative strategies were calculated as $3.7421\times 10^{-4}$ and $2.0380\times 10^{-4}$, respectively.

In [32], the probability β that the adversary does not detect the presence of the embedded message is defined as

(16) $$\beta \geq 2^{-\epsilon }$$

For the proposed system, the probability that the adversary does not detect the presence of the embedded message is β=0.99 for both the additive and multiplicative strategies; thus, the proposed scheme is secure in terms of the Kullback–Leibler divergence test.

### 5.5. Payload

**Figure 14:**
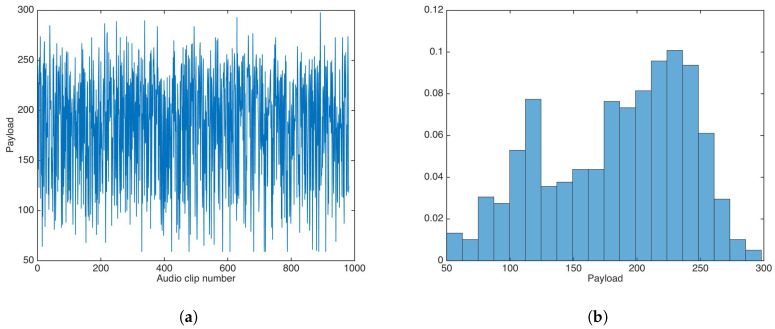
Payload results for the proposed data-hiding scheme. (**a**) Payload results. (**b**) Payload distribution.

Payload is a variable feature in the proposed data-hiding scheme, as it depends of entropy in each sub-band of each audio clip. Figure 14 shows the payload results for the audio dataset.

The payload mean, median, and variance were calculated as 184.54, 195, and 3,088, respectively; the minimum and maximum payload values were 59 and 298, respectively, for 5-s audio clips.

### 5.6. Lossy Compression

Lossy compression is a common signal processing tool in multimedia; for most applications, lossy compression is a requirement as storage space and communications bandwidth must be optimized. The proposed data-hiding scheme was evaluated under Advanced Audio Coding (AAC) audio compression. AAC is used for experimentation, as it has shown better performance in perceptual transparency and compression rates, as compared with Motion Picture Expert Group MPEG-1 and MPEG-2 Audio Layer 3 (MP3) [33]. The experiment was carried out using the multiplicative strategy, as it has been shown (above) to be the best option. The bitrate for compressor was set to 256 kilobits per second (kbps), as this quality is standard for online music distribution.

**Figure 15:**
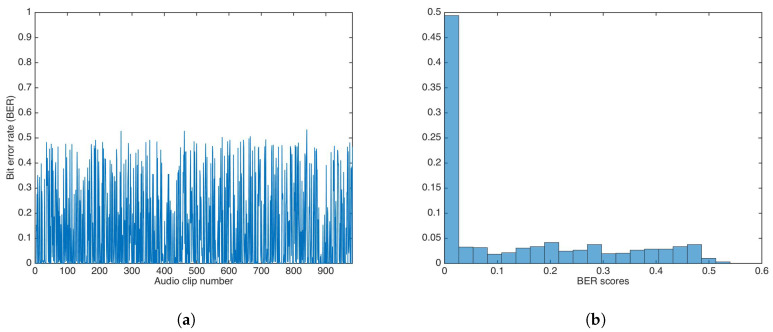
BER results after Advanced Audio Coding (AAC) compression at 256 kbps. (**a**) BER results. (**b**) BER distribution.

Figure 15 shows the BER results and the BER distribution after AAC compression.

The BER median and median absolute deviation (MAD) were calculated as 0.0312 and 0.0312, respectively.

## 6. Discussion and Conclusions

In this paper, we have proposed a data-hiding scheme using sub-band entropy for embedding region selection. The insertion and extraction stages were based on a gain-invariant watermarking approach, previously proposed in [13]. The insertion domain, the intDCT coefficients, was shown be suitable for data-hiding, holding very good auditive transparency. Although the approach in [13] was robust to gain attack and other common (intentional and unintentional) attacks, it lacked key-based security. Two strategies for key-based security—the additive and the multiplicative strategies—were proposed and evaluated. It was shown that both strategies had the same auditive transparency as the proposed data-hiding scheme without key-based security. In the experiments, the PSNR mean and median were calculated as 44.37 and 43.29, respectively, and ODG mean and median were calculated as −0.9362 and −0.6318, respectively, for the unprotected scheme. Thus, additive strategy had PSNR mean and median as 44.36 and 43.30, respectively, and ODG mean and median as −1.0638 and −0.6409, respectively. In similar way, the multiplicative strategy achieved very close values, with PSNR mean and median as 44.34 and 43.29, respectively, and ODG mean and median as −0.9387 and −0.6222, respectively. These results are highly competitive against state-of-the-art schemes based on embedding-region selection by entropy, such as [20,34], for which the best and average PSNR value were 36.4 dB and 37.2 dB, respectively. Table 1 shows a comparison between the proposed data-hiding scheme and the relevant entropy-based audio-watermarking approaches. Statistical tests have shown that there was no significant difference between the unprotected scheme and the key-based protected strategies, in terms of PSNR and ODG; thus, Kerckhoff’s principle was achieved. However, key-based security strategies have differences between them after the Wilcoxon rank-sum test: The multiplicative strategy produced better BER results than the additive strategy when an incorrect key was used for data extraction; both BER mean and median were practically 0.5 for the multiplicative strategy, and were 0.4591 and 0.4582, respectively, for the additive strategy. As can be read in Table 1, only the approach proposed in [34] holds key-based security; however, the complexity of its solution is higher than the proposed strategy in this paper, as the approach in [34] encrypts the data to embed using chaotic mapping; thus, our proposed strategies require a key-generated sequence and addition or multiplication only.

The steganographic capacities of the proposed data-hiding scheme were evaluated using the Kullback–Leibler divergence. Despite very few outliers having been measured in the audio dataset, the median value of KL divergence for both the additive and multiplicative strategies were calculated as $3.7421\times 10^{-4}$ and $2.0380\times 10^{-4}$, respectively; according to the authors of [32], the proposed data-hiding scheme is secure for steganographic applications. From Table 1, it can be observed that only the proposed data-hiding scheme showed statistical transparency; thus, it is attractive for both watermarking and steganographic applications.

On the other hand, payload evaluation results seemed to prove inconclusive, as payload did not follow a clear distribution for the utilized audio dataset, with high payload variance. This behavior may have been due to differences in musical genre or the spectral content of the audio clips; the audio dataset in this study [27] had no explicit music style classification, which makes it difficult to try correlate payload with music genre or spectral content; thus, more research is needed to clarify this behavior. Despite this, for some audio clips, the payload was higher than the average payload in [20].

Finally, a lossy compression evaluation showed that the proposed scheme had adequate performance for current practical applications, as the BER median was 0.0312 after high-quality lossy compression. In [13], the authors analytically showed the performance of their approach under noise and empirically showed the performance under lossy compression using digital images as host signals. The performance of the proposed scheme in this paper confirms the results in [13] for lossy compression; thus, key-based security is guaranteed.

## Figures and Tables

**Table 1:** Comparison of proposed data-hiding scheme and relevant entropy-based approaches.

Reference	ODG	PSNR (dB)	Loosy Compression	Key-Based Security	Statistical Transparency
[20]	n.r. ${}^{1}$	36.4	MP3 (128 kbps)	not	n.r.
[34]	−0.29	37.2	MP3 (128 kbps)	yes	n.r.
Proposed	−0.62	43.2	AAC (256 kbps)	yes	yes

${}^{1}$ not reported.

## References

[1] Kim K., Lee M., Lee H., Lee H.K. (2009). Reversible data hiding exploiting spatial correlation between sub-sampled images. Pattern Recognit..

[2] Nishimura A. Reversible audio data hiding using linear prediction and error expansion. Proceedings of the Seventh International Conference on Intelligent Information Hiding and Multimedia Signal Processing.

[3] Bassia P., Pitas I. (2001). Robust Audio Watermarking in the Time-Domain. IEEE Trans. Multimed..

[4] Ko B.S., Nishimura R., Suzuki Y. (2005). Time Spread Echo Method for Digital Audio Watermarking. IEEE Trans. Multimed..

[5] Kuribayashi M. (2011). Hierarchical Spread Spectrum Fingerprinting Scheme Based on the CDMA Technique. EURASIP J. Inf. Secur..

[6] Arnold M., Cheng X.M., Gries U., Doerr G. (2014). A Phase-based Audio Watermarking System Robust to Acoustic Path Propagation. IEEE Trans. Inf. Forensics Secur..

[7] Kirovski D., Malvar H. (2003). Spread spectrum watermarking of audio signals. IEEE Trans. Signal Process..

[8] Cox I., Kilian J., Leighton T., Shamoon T. (1997). Secure Spread Spectrum Watermarking for Multimedia. IEEE Trans. Image Process..

[9] Chen B., Wornell G. (2001). Quantization index modulation: A class of provably good methods for digital watermarking and information embedding. IEEE Trans. Inf. Theory.

[10] Chen B., Wornell G. (2001). Implementations of quantization index modulation methods for digital watermarking and information embedding of multimedia. J. VLSI Signal Process. Syst. Signal Image Video Technol..

[11] Gonzalez F.P., Mosquera C., Barni M., Abrardo A. (2005). Rational Dither Modulation: A high rate data-hiding method invariant to gain attacks. IEEE Trans. Signal Process..

[12] Garcia-Hernandez J.J., Nakano M., Perez H. (2008). Data Hiding in Audio Signals Using Rational Dither Modulation. IEICE Electron. Express.

[13] Zareian M., Tohidypour H.R. (2014). A Novel Gain Invariant Quantization-Based Watermarking Approach. IEEE Trans. Inf. Forensics Secur..

[14] Guccione P., Scagliola M. (2009). Hyperbolic RDM for nonlinear valumetric distortions. IEEE Trans. Inf. Forensics Secur..

[15] Perez-Gonzalez F., Mosquera C. (2008). Quantization-based data hiding robust to linear-time-invariant filtering. IEEE Trans. Inf. Forensics Secur..

[16] Zhu X., Peng S. A novel quantization watermarking scheme by modulating the normalized correlation. Proceedings of the IEEE International Conference in Acoustic, Speech and Signal Processing.

[17] Lai C.C. (2011). An Improved SVD-based watermarking scheme using visual characteristics. Opt. Commun..

[18] Makbol N.M., Khoo B.E., Rassem T.H. (2016). Block-based discrete wavelet transform-singular value decomposition image watermarking scheme using human visual system characteristics. IET Image Process..

[19] Sangeethaa N., Anitab X. (2018). Entropy based texture watermarking using discrete wavelet transform. Optik.

[20] Chen S.T., Huang H.N., Chen C.J., Tseng K.K., Tu S.Y. (2013). Adaptive audio watermarking via the optimization point of view on the wavelet-based entropy. Digit. Signal Process..

[21] Liu J., Wu S., Xu X. (2018). A Logarithmic Quantization-Based Image Watermarking Using Information Entropy in the Wavelet Domain. Entropy.

[22] Reza F.M. (1961). An Introduction to Information Theory.

[23] Haibin H., Rahardja S., Rongshan Y., Xiao L. A fast algorithm of integer MDCT for lossless audio coding. Proceedings of the IEEE International Conference on Acoustics, Speech, and Signal Processing (ICASSP ’04).

[24] Hore A., Ziou D. Image Quality Metrics: PSNR vs. SSIM. Proceedings of the 20th International Conference on Pattern Recognition (ICPR).

[25] Ismail Avcibas B.S. (1999). Statistical Analysis of Image Quality Measures.

[26] Garcia-Hernandez J.J., Feregrino-Uribe C., Cumplido R., Parra-Michel R. (2010). Improving the Security of Fallahpour’s Audio Watermarking Scheme. IEICE Electron. Express.

[27] Garcia-Hernandez J.J. Replication Data for: “On a Key-Based Secured Audio Data-Hiding Scheme Robust to Volumetric Attack with Entropy-Based Embedding” Submitted to Entropy. https://dataverse.harvard.edu/dataset.xhtml?persistentId=doi:10.7910/DVN/5QSQDL.

[28] Thiede T., Treurniet W., Bitto R., Schmidmer C., Sporer T., Beerens J., Colomes C., Keyhl M., Stoll G., Brandenburg K. (2000). PEAQ—The ITU Standard for Objective Measurement of Perceived Audio Quality. AES.

[29] Kabal P. (2002). An Examination and Interpretation of ITU-R BS. 1387: Perceptual Evaluation of Audio Quality.

[30] Gibbons J.D., Chakraborti S. (2011). Nonparametric Statistical Inference.

[31] Abdi H., Salkind N. (2007). The Bonferonni and Sidak Corrections for Multiple Comparisons. Encyclopedia of Measurement and Statistics.

[32] Cachin C. (2004). An information-theoretic model for steganography. Inf. Comput..

[33] Brandenburg K. MP3 and AAC Explained. Proceedings of the Audio Engineering Society Conference: 17th International Conference: High-Quality Audio Coding.

[34] Dhar P.K., Shimamura T. (2015). Blind SVD-based audio watermarking using entropy and log-polar transformation. J. Inf. Secur. Appl..

